# Latin American Scientific Production on COVID-19 Vaccines

**DOI:** 10.4314/ejhs.v32i2.3

**Published:** 2022-03

**Authors:** Ronald M Hernández, Renzo Felipe Carranza Esteban, Oscar Mamani-Benito, Josué Edison Turpo Chaparro, Miguel A Saavedra-López, Xiomara M Calle-Ramirez, Margarita Wong-Fajardo, Osmer Campos-Ugaz

**Affiliations:** 1 Universidad Católica Santo Toribio de Mogrovejo, Chiclayo, Peru; 2 Universidad San Ignacio de Loyola, Lima, Peru; 3 Universidad de Señor de Sipan, Chiclayo, Peru; 4 Universidad Peruana Unión, Peru; 5 Universidad Continental, Cusco, Peru; 6 Universidad Nacional de Tumbes, Tumbes, Peru

**Keywords:** Scientific production, Vaccine, COVID-19, Latin America

## Abstract

**Background:**

Currently, there is a worldwide health crisis due to the COVID-19 pandemic; consequently, it is necessary to find effective vaccines in order to immunize the population and prevent the transmission of the disease. Likewise, it is important to know vaccine progress and efficacy research, mainly in Latin American countries where no studies have been conducted yet to know the scientific production on COVID-19.

**Methods:**

A retrospective and descriptive study was carried out and COVID-19 vaccine publications in Scopus-indexed journals were considered as a unit of analysis for the period between 2020 and June 2021, with authors affiliated to Latin American institutions.

**Results:**

We found 141 published Scopus-indexed COVID-19 vaccine articles with authors affiliated to Latin American institutions. Brazil has the highest scientific production with 33.33%, followed by Mexico, Colombia, Argentina and Chile. Regarding productivity by institution, 137 international institutions have participated in the Latin American COVID-19 vaccine production. The journals with the highest number of published articles are Vaccines and Vaccine. Both journals are located in Q1 of the SJR. The most frequently used descriptor was coronavirus disease 2019.

**Conclusion:**

The Latin American scientific production on COVID-19 vaccines included 141 published Scopus-indexed articles. Likewise, Brazil is the Latin American country with the highest scientific production.

## Introduction

The world continues experiencing an unprecedented crisis due to the rapid spread of the new coronavirus (COVID-19). More than a year has passed since the beginning of the health emergency and there is no doubt about the revolution that has taken place in the field of research to find a cure, thus, rapid progress has been made in understanding the biology of SARS-CoV-2 and producing safe and effective vaccines (1). Currently, there are alternatives licensed and recommended by the World Health Organization (2).

In this case, an effective vaccine is crucial to prevent morbidity and mortality due to COVID-19. Therefore, the objective in vaccine development is to evidence their efficacy to protect the world's population(3). In this regard, it was known that as of February 2021 there were 44 candidate vaccines in clinical phase and 154 in preclinical pase (4). With respect to their characteristics, most of them target the peak protein (S) of the virus (5), which is found on the surface of the SARS-CoV-2 envelope, allowing it to bind to a cellular receptor (angiotensin-converting enzyme 2) and penetrate host cells. In this regard, studies have shown that activating neutralizing antibodies against the S protein would provide protection against infection. Therefore, the peak protein represents the target of most of the vaccines developed in 2020.

Different technologies are currently being applied in vaccines, also known as platforms. These fall into two categories: whole virus-based and viral protein-based (6). Now, a remarkable issue is the speed with which they were developed, and this is due to different reasons such as the fact that the causative agent was quickly characterized and found to be relatively stable and that the immunity to the coronavirus family was already known, thus, previous research made possible the use of innovative vaccination platforms, and finally, due to the unprecedented scientific and financial deployment that made it possible for clinical trials to be conducted in record time (7). In that sense, platform, adjuvants, form of administration, age and pre-existing crossreactive immunity essentially determine safety, however, due to emergence of resistant variants it is possible that vaccines may need updating.

At this point, it is estimated that the world may return to a pre-pandemic state if a fairly equitable vaccination process is achieved among all countries (8), so it is important to recognize that the strongest predictor of COVID-19 vaccination intentions is to trust in the safety of the vaccines (9), even despite the existence of manageable systemic and local side effects (10). However, there is a large part of the population that does not want to be vaccinated, thus, researchers like Troiano and Nardi (9) studied the most frequent reasons for refusing vaccination, the most noteworthy being concerns about safety, thinking that a hastily produced vaccine is too dangerous, considering that the vaccine is useless due to harmless nature of COVID-19, doubts about the efficacy of the vaccine, doubts about the origin of the vaccine, among others. These facts have motivated researchers to develop measurement instruments such as a scale to measure the perception of SARS-CoV-2 vaccine acceptance (11).

In relation to the above, research results show that Vietnam, India, China, Denmark, South Korea, Serbia, Croatia, France, Lebanon and Paraguay are countries with populations that show a higher proportion of vaccine acceptance(12). However, in many Latin American countries there is still an unfavorable attitude, partly due to the role played by the media(13), and mainly, irrational beliefs and fatalistic ideas resulting from culture and scarce scientific information on vaccine importance (14,15).

In view of these facts, it is extremely important to analyze the scientific production on vaccines. Although the literature contains research that evaluates the scientific activity on COVID-19 in databases such as Scopus(16) and Web of Science (17), there are only vaccine studies that describe the current vaccine platforms(18), the current status of COVID-19 vaccines and therapies (19), but there are no studies that guide researchers on what has already been produced and what is being researched in order to address knowledge gaps through future research.

Therefore, the objective of this study is to analyze the Latin American scientific production on COVID-19 vaccines.

## Materials and Methods

**Study design**: Retrospective descriptive study. The documents that served as source for the analysis came from the Scopus databases, during the period January 2020 to June 2021. The search equation was used to extract documents: “Vaccine” and its relation with the terms: “2019-nCoV” OR “SARS-CoV-2” OR “2019 novel coronavirus” OR “Covid-19” OR “Coronavirus disease 2019” in the fields of title, abstract and keywords considering only those documents that were written by authors mentioning affiliations with Latin American institutions. The variables of author, institution, country and keywords were normalized, since production indicators were generated from them.

**Data collection**: Through this search strategy, 156 documents were retrieved, which were subjected to a process of metadata normalization and elimination of documents that did not deal with the subject. The final sample for analysis was composed of 141 documents.

**Data analysis**: With the extracted documents, a database was organized in Microsoft Excel that included the following data: name of the signing authors, title of the publication, type of publication, affiliation institutions of the signing authors, journal of publication and country of publication. Finally, with the support of the VOSviewer software, a network was created with the main thematic axes associated with the key words of the publications.

## Results

A total of 141 published Scopus-indexed articles with authors affiliated to Latin American institutions were found. Five types of publishable documents were included in the analysis. Original articles represent the largest number of documentary production (n=71; 50.35%), followed by review articles (n=41;29.08%), letters to the editor (n=12;8.51%), editorial (n=12;8.51%) and finally Notes (n=5;3.55%). Vaccine publications during the last year increased by 26.24%.

Brazil is the Latin American country with the highest scientific production on vaccines, representing 33.33% of the Latin American production, followed by Mexico, Colombia, Argentina and Chile, countries with 10 or more publications during the period under evaluation. There are 05 Latin American countries (Dominican Republic, Puerto Rico, Costa Rica, Bolivia and Jamaica) with at least 01 publication (Table 1).

**Table 1:** Latin American countries with scientific production on COVID-19 vaccines

Country	2020		2021	
	
	n	%	n	%
Brazil	13	9.2	34	24.1
Mexico	10	7.1	15	10.6
Colombia	8	5.7	8	5.7
Argentina	4	2.8	10	7.1
Chile	5	3.5	6	4.3
Ecuador	2	1.4	3	2.1
Peru	2	1.4	2	1.4
Cuba	1	0.7	3	2.1
Panama	0	0.0	3	2.1
Uruguay	2	1.4	0	0.0
Venezuela	0	0.0	2	1.4
Paraguay	2	1.4	0	0.0
Dominican Republic	1	0.7	0	0.0
Puerto Rico	0	0.0	1	0.7
Costa Rica	1	0.7	0	0.0
Bolivia	1	0.7	1	0.7
Jamaica	0	0.0	1	0.7
Total	52	36.9	89	63.1

In terms of productivity by institution, 137 international institutions have participated in the Latin American production on Covid-19 vaccines; however, only 02 have produced more than ten articles. Table 3 presents the results of institutions with a publication frequency of five or more papers, among which institutions from Brazil, Colombia, Mexico, Argentina and Chile stand out. Additionally, 30% of these institutions rank in the top 10 of the Ibero-American Ranking of Higher Education Institutions 2020 which classifies institutions according to the number of papers indexed in the Scopus database (Table 2).

**Table 3:** Most productive journals in the topic of COVID-19 vaccines

Journal	Papers	Country	Quartile in SJR	SJR 2020	Categories
Vaccines	9	Switzerland	Q1	1.3	Immunology and Microbiology; Medicine; Pharmacology, Toxicology and Pharmaceutics
Vaccine	7	Holland	Q1	1.59	Biochemistry, Genetics and Molecular Biology; Immunology and Microbiology; Medicine; Veterinary
Frontiers In Immunology	6	Switzerland	Q1	2.65	Immunology and Microbiology; Medicine
Human Vaccines And Immunotherapeutics	6	United States	Q1	1.04	Immunology and Microbiology; Medicine; Pharmacology, Toxicology and Pharmaceutics
Lancet	5	United Kingdom	Q1	13.1	Medicine
ACS Pharmacology and Translational Science	3	United States	Q1	2.27	Medicine; Pharmacology, Toxicology and Pharmaceutics
International Journal of Infectious Diseases	3	Holland	Q1	1.28	Medicine
Nature Medicine	3	United Kingdom	Q1	19.54	Biochemistry, Genetics and Molecular Biology; Medicine
Applied Health Economics and Health Policy	2	Switzerland	Q1	1.1	Economics, Econometrics and Finance; Medicine
Boletín Médico Del Hospital Infantil De México	2	Mexico	Q4	0.17	Medicine

**Table 2:** Latin American Institutions participating in COVID-19 vaccine research

Institution	Country	Place in SIR Iber 2020	Papers
Universidade de Sao Paulo -USP	Brazil	1	11
Fundacion Universitaria Autónoma de Las Americas	Colombia	604	11
Universidad Nacional Autónoma de México	Mexico	2	9
Universidad Tecnológica de Pereira	Colombia	235	9
Universidade Federal do Rio de Janeiro	Brazil	9	8
Consejo Nacional de Investigaciones Científicas y Técnicas	Argentina	-	8
Fundacao Oswaldo Cruz	Brazil	-	7
Universidade Federal de Sao Paulo	Brazil	24	5
Pontificia Universidad Católica de Chile	Chile	25	5
Universidade Federal de Minas Gerais	Brazil	13	5

Table 3 lists the most productive journals, among which Vaccines and Vaccine stand out (more than 7 papers published). Both journals are located in Q1 of the SJR. Scientific production is concentrated in European and North American journals, and 90% of such journals are located in Q1. This demonstrates not only high visibility of the contributions but also their possible quality.

Table 4 shows the authors with the highest number of COVID-19 vaccine studies to date. Only one author (Rodriguez-Morales, Alfonso J.) out of the 121 authors of the 141 papers analyzed has consistently produced (n=9) papers to date.

**Table 4:** Latin American authors with the highest production of COVID-19 vaccine papers

Author	Institution	Country	H- Index	Papers
Rodriguez-Morales, Alfonso J.	Fundación Universitaria Autónoma de las Américas	Colombia	42	9
Cerda, Arcadio Alberto Cerda	Universidad de Talca	Chile	5	4
García, Leidy Y.	Universidad de Talca	Chile	4	4
Bonilla-Aldana, D. Katterine	Fundación Universitaria Autónoma de las Américas	Colombia	17	3
Iqbal, Hafiz M.N.	Tecnológico de Monterrey	Mexico	48	3
Kalergis, Alexis Mikes	Pontificia Universidad Católica de Chile	Chile	40	3

The ten most cited COVID-19 vaccine articles are presented. Eighty percent of them were published in 2020 and only two of the total number have been cited more than 100 times (Table 5).

**Table 5:** Most cited Latin American-authored COVID-19 vaccine articles

Title of paper	Journal	Year of publication	Citations in Scopus
Safety and efficacy of the BNT162b2 mRNA Covid-19 vaccine	New England Journal of Medicine	2020	697
Safety and efficacy of the ChAdOx1 nCoV-19 vaccine (AZD1222) against SARS-CoV-2: an interim analysis of four randomised controlled trials in Brazil, South Africa, and the UK	The Lancet	2021	304
Nanotechnology for COVID-19: Therapeutics and Vaccine Research	ACS Nano	2020	54
An ethical framework for global vaccine allocation	Science	2020	47
A Single Immunization with Nucleoside-Modified mRNA Vaccines Elicits Strong Cellular and Humoral Immune Responses against SARS-CoV-2 in Mice	Immunity	2020	38
Single-dose administration and the influence of the timing of the booster dose on immunogenicity and efficacy of ChAdOx1 nCoV-19 (AZD1222) vaccine: a pooled analysis of four randomised trials	The Lancet	2020	36
Contingent assessment of the COVID-19 vaccine	Vaccine	2020	24
SARS-CoV-2/COVID-19 and advances in developing potential therapeutics and vaccines to counter this emerging pandemic	Annals of Clinical Microbiology and Antimicrobials	2020	22
COVID-19 - Recent advancements in identifying novel vaccine candidates and current status of upcoming SARS-CoV-2 vaccines	Human Vaccines and Immunotherapeutics	2020	22
What does plant-based vaccine technology offer to the fight against COVID-19?	Vaccines	2021	21

**Figure 1:**
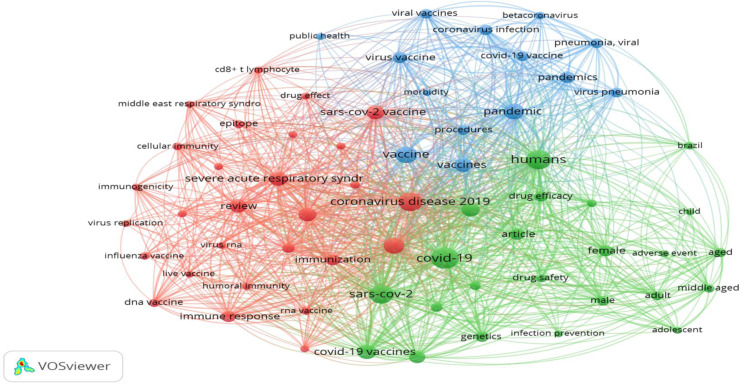
Visualization of keyword occurrence network

Figure 1 shows the most frequent descriptors, with coronavirus disease 2019 having the highest number of occurrences (n=64). The number of word co-occurrences indicates the number of publications in which they appear in the selected documents and the colors indicate clusters of keywords relatively related to each other according to the strong association obtained by the VOSviewer program, in addition to the visual difference of clusters. With the 65 descriptors out of a total of 1773 descriptors recorded in the 141 documents retrieved and the three clusters, the thematic focus of each cluster was analyzed. Cluster 1 (red) analyzes the relationship between COVID-19 and factors conditioning immune response. Cluster 2 (green) analyzes the themes on COVID-19 and the safety and efficacy of the drugs provided. The last cluster (blue) describes the COVID-19 vaccine to mitigate the pandemic.

## Discussion

Coronavirus disease 2019 (COVID-19) has caused millions of deaths and economic and social crises worldwide(8), and different research centers around the world have published their results as the first COVID-19 vaccine candidates, representing a source of hope for the international community (4,7). However, it is also important to know related research from other parts. Latin America is one of the regions most affected by COVID-19 in terms of cases and deaths, in addition to seriously affecting individuals' mental health (20). In this context, the objective of this research was to analyze the Latin American scientific production on COVID-19 vaccines.

Our results confirm that Latin America produced a total of 141 COVID-19 vaccine articles. This result compared with the world production of COVID-19 vaccine articles, which is 2,625 articles, where only Brazil appears in 15th place, far from the production of other countries such as China, the USA, the United Kingdom and Italy (17). The results are similar to those found in a study on the scientific production of mental health in the time of Covid-19, where the Latin American scientific production was 4.74% of the world production, which shows the scarce publication in Latin America (21).

Likewise, the bibliometric analysis carried out through this study has made it possible to characterize these researchers during the pandemic. Brazil has contributed the largest number of scientific publications on vaccines. This can be explained by the fact that Brazil hosts the largest number of top Latin American research centers and universities (22), and that Brazil is the country most affected by Covid-19(23) and despite the fact that its government has shown little importance in this area(24). Mexico occupies second place in the ranking. This can be explained by the fact that Mexico is the country with the second largest population (20) and also that together with Brazil they are leaders in scientific development and production at the Latin American level (25) and in Covid-19 production (26,27).

It is also important to highlight a significant number of authors who have taken advantage of the opportunity given by the pandemic and the importance of this to increase the number of studies published through letters to the editor, review articles and original articles (17).

With respect to the most cited journals in Scopus, Vaccines, Vaccine and Frontier In Immunology ranked 1^st^, 2^nd^ and 3^rd^, respectively. This could be due to the fact that they are journals of global scope, with a high impact factor, and with open calls for manuscripts on this topic. Additionally, as in the case of Vaccine, it is an important publication for Latin America (24). All these journals have a great scientific influence and although due to the speed of publications some retractions were found(26), they remain in the world top, which means a greater number of citations(27).

With an H-Index of 42, the most published author is Alfonso Rodriguez-Morales, Colombian, with 9 articles. He is among the authors who have also contributed the most number of COVID-19 studies(25). In terms of productivity by institutions, both USP (Brazil) and the Fundación Universitaria Autónoma de Las Américas (Colombia) lead in this category. It should be noted that the author Rodriguez-Morales belongs to the Colombian institution.

With 697 citations in Scopus, the published co-authored article with the highest impact was by Polack, Thomas, Kitchin et al. (28) that was published in The New England Journal of Medicine. This article aimed to report safety and efficacy findings from the phase 2/3 part of a global trial evaluating the safety, immunogenicity, and efficacy of the BNT162b2 mRNA COVID-19 vaccine for the prevention of COVID-19 disease in individuals aged 16 and older. The authors demonstrated that a two-dose regimen of BNT162b2 conferred 95% protection against COVID-19 in individuals aged 16 or older. In a bibliometric investigation of rabies vaccination over the past three decades, they concluded the importance of strengthening collaborative research between institutions and researchers in developing countries and developed countries (29).

Another key factor was the co-occurrence relationships between keyword pairs that were determined from the number of articles in the database. Cluster 01 (red) relates to research addressing the epidemic and factors conditioning immune response. Cluster 02 (green) analyzes the safety and efficacy of COVID-19 treatments. Cluster 03 (blue) highlights the COVID-19 vaccine characteristics to mitigate the epidemic. In a study conducted on Covid-19 vaccine research, the most used keywords were COVID and vaccine; also according to visualization mapping COVID-19 was the most co-occurring author keyword (30).

The findings of this research show that bibliometric studies characterize research conducted in Latin America. Through this design, the typology of publication, institutions, researchers, number of citations of scientific production are observed. In addition, the most analyzed groups with respect to the topic of COVID-19 vaccines are observed.

However, this bibliometric study has limitations. Only the Scopus database was used. Although it is a referential platform with broad coverage in science, we believe it is necessary to conduct similar research using other databases through additional studies. It is also important to note that the number of publications and citations (31) of co-authorship is increasing every day, so it is very difficult to keep a daily control, which we also consider to be a limitation. Therefore, the researchers determined a time frame for data collection and performing their research and discussion. It is possible that data may have been lost in the course of this process and that reality does not coincide with the data shown in this research.

Despite these limitations, we consider that this research contributes to the global thematic on the Covid-19 vaccine, finding articles published in different journals indexed in Scopus, which have links to institutions located in the top 10 of the Ibero-American ranking of Higher Education Institutions located in 17 Latin American countries.
